# The phenomenology of pareidolia in healthy subjects and patients with left- or right-hemispheric stroke

**DOI:** 10.1016/j.heliyon.2024.e27414

**Published:** 2024-02-29

**Authors:** M. Camenzind, N. Göbel, A.K. Eberhard-Moscicka, S.E.J. Knobel, H. Hegi, M. Single, B.C. Kaufmann, R. Schumacher, T. Nyffeler, T. Nef, R.M. Müri

**Affiliations:** a Perception and Eye Movement Laboratory, Departments of Neurology and BioMedical Research, Inselspital, Bern University Hospital and University of Bern, Switzerland; b Research and Analysis Services, University Hospital Basel and University of Basel, Basel, Switzerland; c Department of Neurology, Inselspital, Bern University Hospital, University of Bern, Switzerland; d Department of Psychology, University of Bern, Bern, Switzerland; e Gerontechnology and Rehabilitation Group, ARTORG Center for Biomedical Engineering Research, University of Bern, Bern, Switzerland; f Neurocenter, Luzerner Kantonsspital, Lucerne, Switzerland

**Keywords:** Pareidolia, Hemispheric stroke, Visual processing, Visual illusions, Natural images

## Abstract

Pareidolia are perceptions of recognizable images or meaningful patterns where none exist. In recent years, this phenomenon has been increasingly studied in healthy subjects and patients with neurological or psychiatric diseases. The current study examined pareidolia production in a group of 53 stroke patients and 82 neurologically healthy controls who performed a natural images task. We found a significant reduction of absolute pareidolia production in left- and right-hemispheric stroke patients, with right-hemispheric patients producing overall fewest pareidolic output. Responses were categorized into 28 distinct categories, with ‘Animal’, ‘Human’, ‘Face’, and ‘Body parts' being the most common, accounting for 72% of all pareidolia. Regarding the percentages of the different categories of pareidolia, we found a significant reduction for the percentage of “Body parts” pareidolia in the left-hemispheric patient group as compared to the control group, while the percentage of this pareidolia type was not significantly reduced in right-hemispheric patients compared to healthy controls. These results support the hypothesis that pareidolia production may be influenced by local-global visual processing with the left hemisphere being involved in local and detailed analytical visual processing to a greater extent. As such, a lesion to the right hemisphere, that is believed to be critical for global visual processing, might explain the overall fewest pareidolic output produced by the right-hemispheric patients.

## 1 Introduction

The phenomenon of pareidolia is defined as the perception of a recognizable image or a meaningful pattern where none exists or is intended, such as the perception of an animal in surface features, or a tendency to interpret a vague stimulus as something known to the observer. The word pareidolia derives from two Greek words, i.e., para παρά (beside, alongside, instead) and eidōlon, εἴδωλον (image, form, shape).

For many decades, pareidolia were considered more of a curiosum with some prominent examples making the headlines. These include the face of Jesus in a toast, a cinnamon bun with the face of Mother Teresa, or Satan appearing in the smoke of the World Trade Center of 9/11 [1,2]. Only in recent years, the scientific value of pareidolia as a possible biomarker of different clinical conditions in neurology and psychiatry was emphasized. One of the pioneering papers by Uchiyama et al. (2012) [3] explored the clinical utility of pareidolia in patients with dementia with Lewy bodies and Alzheimer's disease. This influential study found that patients with dementia with Lewy bodies produced a significantly greater number of pareidolia compared to patients with Alzheimer's disease or controls. Notably, the categories of “Figures”, “Faces of people”, and “Animals” accounted for over 80% of the contents of the pareidolia. Other clinical studies also focused on face pareidolia but employed different images and experimental instructions (e.g., [[4], [5], [6]]). Since pareidolia may represent subclinical hallucinations or a predisposition to visual hallucinations, pareidolia were also tested in patients with Parkinson's disease (e.g., [7,8]). In our own study [8], we found an increased number of face-pareidolia in Parkinson's disease patients compared to healthy controls. This finding was especially prominent for Parkinson's disease patients with cognitive impairment.

Face pareidolia in healthy subjects are relatively well studied (e.g. [9,10]). For example, in an fMRI study examining the neural network of face pareidolia, healthy subjects were instructed to search for faces or letters in pure noise images, while they were told that 50% of the images contained either faces or letters [9]. This study found activation of the right fusiform face area (rFFA) when subjects reported seeing faces. The whole brain analyses revealed a face pareidolia network including both frontal as well as occipito-temporal regions. It has been further indicated that individual factors may explain why some healthy subjects perceive more pareidolia than others do (for a review, see Ref. [11]). Zhou and Meng (2020) identified four main factors i.e., sex, developmental factors, personality traits and neurodevelopmental conditions that may influence the individual production of face pareidolia in healthy subjects.

Furthermore, in our own study we investigated the association between pareidolia and creative behavior [12]. We found that divergent thinking, in terms of fluency in the Alternative Uses Task (AUT) was predictive of fluency and originality, suggesting that pareidolia may represent a possible way to investigate divergent aspects of creative cognition. A recent study examining the perceptual aspect of creativity by means of a cloud-like fractal image paradigm confirmed these results [13]. More specifically, they found that pareidolia perceptions arise more rapidly and more often in highly creative individuals.

While the recent years brought many studies investigating pareidolia, its phenomenology is still poorly understood, and the current state of literature leaves open questions. Many of the above-mentioned studies employed instructions that provoked a specific category of pareidolia (e.g., face pareidolia), leaving the entire possible spectrum of pareidolia production unexplored. In order to induce a large spectrum of pareidolia, the instruction shall not only be open, but also the stimulus presentation time shall be long enough, to our opinion. In our study, we were interested whether stroke influences pareidolia production in a similar manner as neurodegenerative diseases do. Furthermore, the extent to which the lesioned hemisphere may influence pareidolia production is to date unknown. Given that the left hemisphere is devoted to more detailed, analytical visual processing, while the right hemisphere is considered to be of a greater importance with respect to global visual processing [14,15] we hypothesized that left- and right-hemispheric lesions may affect visual processing of pareidolia differently. Thus, our research questions were: 1) do stroke patients produce more or rather less pareidolia compared to healthy controls, 2) is there an association with clinical characteristics of the patients, 3) do stroke patients perceive the same pareidolia categories as controls, and 4) do we observe differing patterns depending on the hemispheric lesion side?

## 2 Materials and methods

### 2.1 Participants

Table 1 shows the demographic data of the patient group. Patients were recruited in three rehabilitation clinics, in Bern, Riggisberg, and Lucerne. Patients were evaluated at admission by 2 of the authors (RMM and TN). Inclusion criteria were first-ever hemispheric stroke, age between 18 and 90 years, fluency in either Swiss or High German, and normal or corrected-to-normal visual acuity, and approvement of the patient for the study. Exclusion criteria were global aphasia, complete hemianopia and patients with psychiatric or neurological diseases.

**Table 1:** Demographic data of the patient group.

Side of stroke	Number (N)	Gender	Handedness	Age (mean)	Initial NIHSS	Inclusion after stroke (mean)
Left	N:23	9f	21 right-handed	57 years (SD:13.2)	8 (SD:28)	1.2 months (SD:0.7)
Right	N:30	10f	28 right-handed	61 years (SD:12.5)	9 (SD:6.3)	1.5 months (SD:1.1)

f: female; SD: standard deviation.

Healthy subjects were recruited via various channels (e.g. notice boards, personal contact, or social media).

On average, 12% of the study population was left-handed, which is expected for unbiased samples. Moreover, the Chi-Squared test indicated no significant group differences (Χ^2^ [2] = 1.599, p = 00.450) for handedness.

Fifty-four percent of the left-hemispheric patients presented with right hemisyndrome and 50% of the right-hemispheric patients with left hemisyndrome. Incomplete visual field defects were found in 25% of the left-hemispheric patients and in 10% of the right-hemispheric patients. Sixty-seven percent of left-hemispheric patients had aphasia. Hemineglect was found in 13% of left-hemispheric patients and in 31% percent of right-hemispheric patients. Furthermore, we used TAP-Test of Attentional Performance (Zimmermann and Fimm, PsyTest,^@)^, the subtests Alertness and Go-Nogo to measure alertness and inhibition. The study followed the STROBE guidelines for reporting observational studies and was conducted in accordance with the principles laid down in the Declaration of Helsinki (WHO, 2013).

The study was approved by the local Ethics Committee of the Canton of Bern and by the Ethics Committee of Central Switzerland.

### 2.2 Experimental design

**Fig. 1:**
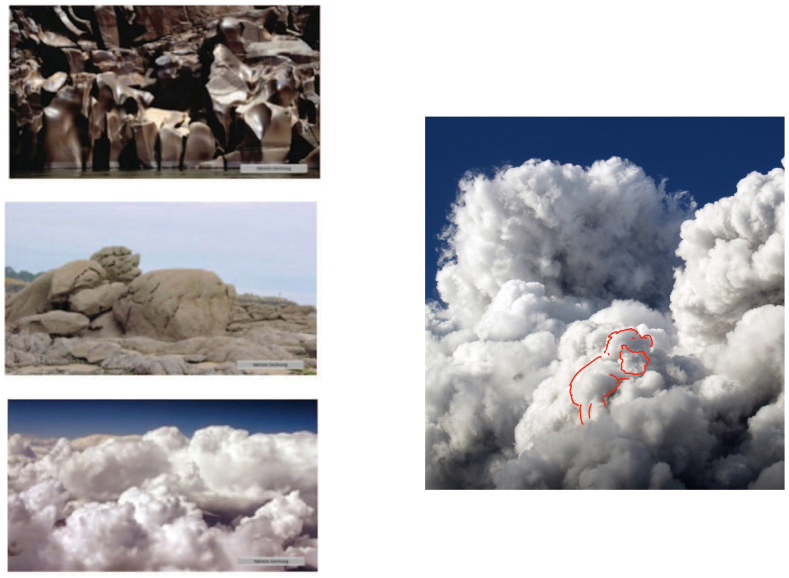
Exemplary natural images used in the study.Left panel: An example set of natural images [12]. All experimental stimuli were unmodified landscapes that purposely did not include animals, objects or human beings. Right panel: An example of a participant's pareidolia drawing (in red). (For interpretation of the references to color in this figure legend, the reader is referred to the Web version of this article.)

For each participant, a set of three natural images (Fig. 1, left panel) was used to prompt pareidolia. Four different image sets were employed in the study, each of which consisted of three unmodified natural images that were presented in a random order for 5 min. The task was programmed in a development platform Unity 2019.3 (Unity Software Inc., San Francisco, United States) and the experimental images were displayed on a Lenovo Yoga tablet 720-15IKB (15.6 Zoll, i7, 16 GB RAM).

The task instruction was “Look at the following pictures and let your imagination run free, draw everything you can see, except the objects themselves (e.g., clouds), and say every time what it is.” Study participants were provided with a compatible digital pen (Lenovo active Pen). The verbal answers were recorded by means of a voice recorder (Jabra PHS001U). These verbal answers were subsequently transcribed by the examiner and further classified in semantic categories. The number of pareidolia produced was counted offline for each participant, and the answers were classified into 28 categories (see section 2.3. below). Prior to the start of the experimental trials each participant was presented with the same training example of a natural image. The training image consisted of a photograph of a heavily weathered piece of wood, like those of the experiment. To demonstrate the task, the instructor sketched some pareidolia and then, let draw the participant some pareidolia.

### 2.3 Statistical analysis

Statistical analyses were performed with JASP Version 0.16.1 (JASP Team, University of Amsterdam, 2022).

The absolute number of pareidolia produced was compared between the three groups by means of Kruskal-Wallis test and the post-hoc Mann-Whitney tests. Bonferroni correction was applied for the post-hoc Kruskal-Wallis tests conducted for the five most frequent categories (Table 2).

**Table 2:** Non-significant influences on pareidolia production of presence or absence of clinical characteristics.

	Present (N)	Absent (N)	U Statistics	P value
	Median (M)	Median (M)		
Upper limb paresis	N: 40	N: 13	253.00	0.60
	M: 21.5	M: 17		
Visual hemineglect	N:12	N: 41	236.50	0.86
	M: 21.5	M: 21		
Visual field defects	N: 9	N: 44	156.50	0.33
	M: 22	M: 19		
Aphasia	N: 19	N: 34	327.50	0.94
	M: 20	M: 22		

N: Number of patients; M: Median number of pareidolia produced.

A post-hoc power analysis was conducted to evaluate the statistical power of the study. This was necessary due to the absence of prior empirical data in stroke patients for a priori power estimation. We used an F test approximation for ANOVA with fixed effects in a one-way design, considering the nonparametric nature of our primary tests. The effect size was estimated at 0.4, the alpha error at 0.05, and the total sample size conservatively at 69, thrice the size of the smallest group. This analysis yielded an actual power of 0.83, suggesting that the study was adequately powered to detect the estimated effect sizes.

## 3 Results

**Fig. 2:**
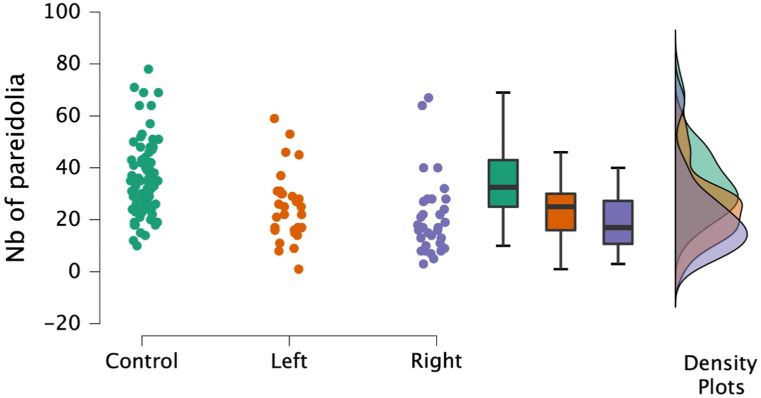
Number of pareidolia production per experimental group.

Overall, participants produced a total of 4097 pareidolia. In the control group the median number of pareidolia was 32.50 (IQR: 18.00), in the left-hemispheric stroke group the median was 23.50 (IQR: 15.00), and in the right-hemispheric stroke group the median was 17.50 (IQR: 14.75, see Fig. 2). Kruskal-Wallis test indicated a significant difference between the groups (H(2) = 26.067, p < 0.001). The post-hoc Mann-Whitney tests revealed significant differences between the control and the left-hemispheric group (U = 9.148 p = 0.002) and between the control and the right-hemispheric group (U = 22.302, p < 0.001). There was no significant difference between the two stroke patient groups (U = 1.323, p = 0.250).

Correlations: There was no significant difference in pareidolia production between right- and left-handed stroke patients (U = 84.00 p = 0.61), and the correlation between initial NIHSS and number of pareidolia was not significant (Pearson's r = 0.04; p = 0.078). The correlations between T-values of the TAP subtests Alertness and Go-Nogo and the number of pareidolia revealed no significant correlations: Pearson's r for T-values of median RT with acoustic cue was 0.1919 p = 0.1774 p; without acoustic cue Pearson's r = 0.2649, p = 0.0603. Pearson's r for median RT in the Go-Nogo subtest was −0.078, p = 0.5901, and for errors, Pearson's r was −0.0596, p = 0.6808. Furthermore, there was no significant correlation for age and pareidolia production (Pearson's r = −0.0205, p = 0.8831.

The statistical comparison between absence and presence of clinical characteristics is shown in Table 2. There was no significant difference in pareidolia production for the presence or absence of upper limb paresis, visual hemineglect, visual field defect or aphasia. There was a trend for the difference in pareidolia production between male and female patients (for females 27 pareidolia (SD:13.7 pareidolia) and for males 22 pareidolia (SD: 15.1 pareidolia); U = 428.5 p = 0.06).

**Fig. 3:**
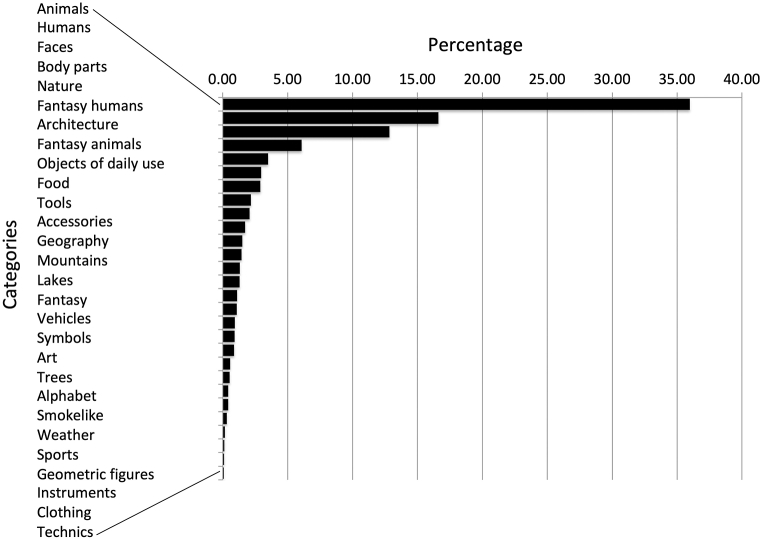
Percentages of pareidolia production accounting for 28 distinct categories.

**Fig. 4:**
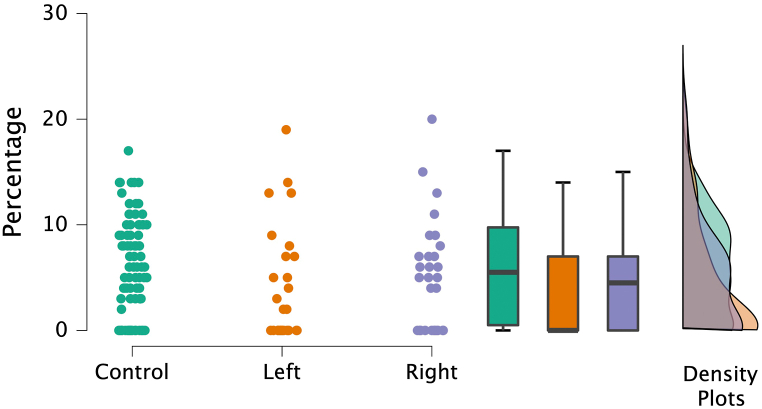
Percentages for the category “Body parts” for the three experimental groups.

Classification of the produced pareidolia resulted in 28 semantic categories (see Fig. 3). Four categories (i.e., “Animals”, “Humans”, “Faces”, and “Body parts”) accounted for 72% of all pareidolia produced. All other 24 categories each accounted for less than 5% of the entire output and were thus pooled into a category “Others” for further statistical analysis (Table 3). Whereas no differences between the three experimental groups were found for the categories “Animals”, “Humans”, “Faces”, and “Others”, significant differences were found for the category “Body parts” (i.e., where subjects perceived a pareidolia such as e.g., a hand, a leg or other parts of the body; H(2) = 7.875, p = 0.019; see Fig. 4 and Table 2). Moreover, the post-hoc Mann-Whitney test conducted for the category “Body parts” indicated a significant difference between the control and the left-hemispheric group (U = 9.148, p = 0.002), the difference between the control and the right-hemispheric group was not significant (U = 1.360, p = 0.243), supporting the hypothesis of hemisphere-specific effects on pareidolia production. Of note, although statistical testing did not reveal significant differences between left- and right-hemispheric patients for the “Body part” pareidolia (U = 2.213, p = 0.137), there may be a tendency.

**Table 3:** Percentages of the pareidolia produced for the four most frequent categories and the category “Others” (i.e., the sum of the 24 pareidolia categories, each of which accounted for less than 5% of the entire output).

	“Animals”	“Humans”	“Faces”	“Body parts”	“Others”
	M (IQR)	M (IQR)	M (IQR)	**M (IQR)**	M (IQR)
Control	34.50 (16.00)	15.50 (11.00)	12.00 (9.00)	**5.5 (9.25)**	26.00 (15.75)
Left	37.00 (28.75)	14.50 (11.25)	12.50 (12.00)	**0.0 (4.25)**	28.00 (26.25)
Right	34.50 (20.50)	13.00 (15.75)	13.50 (18.25)	**5.00 (7.00)**	29.00 (20.50)
Test statistics	H = 0.093 p = 0.955	H = 1.450 p = 0.484	H = 0.038 p = 0.981	**H = 7.88 p = 0.02**	H = 0.652 p = 0.722

M: Median number of pareidolia produced; IQR: interquartile range.

## 4 Discussion

The aim of the current study was to describe the phenomenology of pareidolia in healthy subjects and patients with right- and left-hemispheric stroke. To this end we used an open instruction, and the natural images were presented for 5 min. We found that under these conditions, participants produced 28 different semantic categories of pareidolia. There were substantial differences in their frequencies, merely four categories accounted for 72% of all pareidolia. Notably, “Animal” pareidolia were perceived most often, followed by “Human”, “Face”, and “Body parts” pareidolia. Each of the remaining 24 categories contributed to less than 5% of the entire pareidolia produced.

One of the hallmark studies in clinical pareidolia research is the study by Uchiyama et al. (2012) [3] in which they investigated pareidolia production by means of 25 colored scenery pictures that contained animals, plants and artefacts in patients with dementia. In contrast to the stimuli we used, their 25 experimental stimuli were filtered by Gaussian blurring and presented to participants for 1 min. Their controls perceived no pareidolia in this experimental setting. This suggests that pareidolia production in healthy subjects may be dependent on perceptual properties of the images and on the presentation time. Indeed, longer presentation time may activate visual imagination to a greater extent. We found a significant reduction of pareidolia production both in left- and right-hemispheric stroke patients suggesting that stroke patients in the post-acute phase may have a generally reduced production of pareidolia. Given that cognitive impairment is a commonly reported consequence of stroke with the estimated rates of 35–70% in the post-acute and chronic phases [16], one may argue that the phenomenon of the reduced pareidolia production may be due to post-stroke cognitive deficits. However, there is little consensus as to the true profile of the domain-specific cognitive deficits in stroke populations [17]. Moreover, such a profile varies depending on a variety of factors such as vascular territory [18], lesion side [19], stroke subtype [20], or anatomic localization of the stroke lesion [21]. We found no significant influence of clinical or epidemiological parameters such as age, gender, handedness, initial NIHSS, presence or absence of aphasia, visual field deficit, hemineglect, or upper limb paresis on pareidolia production. Furthermore, alertness or inhibition in the Go-Nogo task did not correlate with pareidolia production in our subacute stroke patients.

Deficits in information processing speed (IPS) are also a common phenomenon in stroke patients. As such, in the acute phase of stroke, up to 70% of stroke survivors may experience slow IPS [22]. Yet IPS is not a stand-alone domain but rather a key cognitive resource that influences higher-order domains such as attention, memory, and executive functioning. Thus, IPS may be a reason for the general reduction of pareidolia production in the group of our stroke patients.

Furthermore, the percentages of “Animal”, “Human”, and “Face” pareidolia were not significantly different between the three groups, suggesting that left- or right hemispheric lesions did not affect the production of the main pareidolia categories per se. With respect to the hemispheric side affected, the only difference we found concerned the “Body parts” pareidolia. While a significant reduction for the percentage of the “Body parts” pareidolia was found in the left-hemispheric patient group as compared to the control group, the percentage of this pareidolia type was not reduced in right-hemispheric patients. This result supports our hypothesis that pareidolia production may be influenced by local-global visual processing with the left hemisphere being involved in local and detailed analytical visual processing to a greater extent. Indeed, functional imaging studies have shown that healthy participants tend to show a right-hemispheric preference in processing global stimuli and a left-hemispheric preference in processing local stimuli [[23], [24], [25]]. Also, studies in brain-damaged patients with unilateral left-hemispheric lesions reported similar deficits [26,27].

One might further expect that the right hemisphere superiority for face perception may further influence the production of “Face” pareidolia, resulting in a reduction of “Face” pareidolia produced following a right-hemispheric lesion. However, the current study did not find support for this speculation as “Face” pareidolia production was not significantly different between the three experimental groups. No significant differences were also found for the categories “Animal”, “Humans”, and “Others”.

In the clinical literature concerning pareidolia in neurodegenerative diseases, visual hallucinations and pareidolia may share similar characteristics. As such, Uchiyama et al. (2012) [3] found that pareidolia categories were consistent with those of visual hallucinations in their patient groups with dementia. Moreover, in their study, illusions of humans and animals accounted for more than 80% of pareidolia. While faces and full-length figures of humans and animals were clearly dominant, other body parts or man-made artefacts were infrequent. Urwyler el al. (2016) [28], who examined complex visual hallucinations in patients with eye diseases affecting visual acuity and patients with Parkinson's disease as well as patients with Lewy body dementia, found that visual hallucinations concerning humans were the most frequent in all three clinical groups. Those hallucinations were followed by hallucinations of animals and body parts in the Parkinson's disease and Lewy body dementia groups. Importantly, the group of patients with complex hallucinations and eye disease differed from the Parkinson's disease and Lewy body dementia group such that the second and the third most common hallucinations concerned body parts and flowers. With respect to this finding, patients and healthy controls assessed in our study, who did not experience hallucinations, exhibited a different pattern, suggesting that pareidolia may not necessarily reflect a simple “precursor” of complex visual hallucinations.

To the best of our knowledge, this is the first study employing natural images in a study of pareidolia production in patients with subacute hemispheric stroke. Our study presents some important differences as compared to previous literature: firstly, the images presented in the current study depicted unmodified natural landscapes; secondly, each image was presented for 5 min; thirdly, we did not employ a classical search task (such as the search face task) but we left the instruction fairly open, i.e., “let your imagination run free”; and lastly, participants were instructed to draw what they perceived. Importantly, only few studies accounted for categories of the pareidolia produced (e.g., [[3], [11], [15]]).

Our study entails some limitations. Given that the current study focused on the phenomenology of pareidolia, subacute stroke patients with lesions in all three arterial territories on both hemispheres were recruited and included in the analysis. Due to this heterogeneity of our population, we were able to demonstrate a broad spectrum of pareidolia categories found in stroke. However, speculations about the neural circuits or regions involved in pareidolia production would not be reliable, and our approach is not suitable for identifying confounding factors.

## 5 Conclusions

This study sought to understand the effect of left- and right hemispheric stroke on pareidolia production. Compared to the healthy control group, stroke patients exhibited a reduced production of pareidolia and an effect of the “Body part” pareidolia suggesting that local-global visual processing may play a role in the production of pareidolia. Overall, however, the general pattern of pareidolia was similar between the stroke patients and the healthy controls. Future research should be focused on the influence of neuroplasticity on pareidolia production during recovery of stroke.

## Funding

This research was funded by the 10.13039/501100001711Swiss National Science Foundation (SNSF), 10.13039/501100001711SNF grant number SNF-175615 to R.M.M.

## Data availability statement

The complete set of images and the data supporting the conclusions of this study will be made available by the corresponding author upon a reasonable request.

## CRediT authorship contribution statement

**M. Camenzind:** Writing – review & editing, Methodology, Investigation, Formal analysis, Data curation. **N. Göbel:** Writing – review & editing, Methodology, Investigation, Formal analysis, Data curation. **A.K. Eberhard-Moscicka:** Writing – review & editing, Writing – original draft, Methodology, Formal analysis, Data curation, Conceptualization. **S.E.J. Knobel:** Writing – review & editing, Methodology. **H. Hegi:** Writing – review & editing, Methodology. **M. Single:** Writing – review & editing, Methodology. **B.C. Kaufmann:** Writing – review & editing, Methodology. **R. Schumacher:** Writing – review & editing, Methodology. **T. Nyffeler:** Writing – review & editing, Supervision, Methodology, Conceptualization. **T. Nef:** Writing – review & editing, Methodology, Conceptualization. **R.M. Müri:** Writing – review & editing, Writing – original draft, Project administration, Methodology, Funding acquisition, Conceptualization.

## Declaration of competing interest

The authors declare that they have no known competing financial interests or personal relationships that could have appeared to influence the work reported in this paper.
